# Adaptive data-driven models to best predict the likelihood of live birth as the IVF cycle moves on and for each embryo transfer

**DOI:** 10.1007/s10815-022-02547-4

**Published:** 2022-06-29

**Authors:** Véronika Grzegorczyk-Martin, Julie Roset, Pierre Di Pizio, Thomas Fréour, Paul Barrière, Jean Luc Pouly, Michael Grynberg, Isabelle Parneix, Catherine Avril, Joe Pacheco, Tomasz M. Grzegorczyk

**Affiliations:** 1 Department of Assisted Reproductive Technology and Fertility Preservation, Clinique Mathilde, Service Assistance Médicale à la Procréation, 76100 Rouen, France; 2 Nantes Université, Inserm, Centre de Recherche en Transplantation Et Immunologie, UMR 1064, ITUN, 44000 Nantes, France; 3 Service de Médecine Et Biologie du Développement Et de La Reproduction, Nantes, France; 4 Centre de Procréation Médicalement, Assistée Hôpital Estaing-CHU de Clermont Ferrand, 63003 Clermont Ferrand, France; 5 Department of Reproductive Medicine and Fertility Preservation, Hôpital Antoine Béclère, Hôpitaux Universitaires Paris Sud, Assistance Publique-Hôpitaux de Paris, 92140 Clamart, France; 6 Department of Reproductive Medicine & Fertility Preservation, Hôpital Jean Verdier, 93140 Bondy, France; 7 Université Paris-Sud, Université Paris Saclay, Le Kremlin Bicêtre 94276, France; 8 Department of Cytogenetic and Reproductive Biology, Hôpital Jean Verdier, 93140 Bondy, France; 9 Unité Inserm U1133, Université Paris-Diderot, 75013 Paris, France; 10 Centre AMP Eurofins Fertilité Jean Villar, Bordeaux, France; 11 Teranalytics, Artificial Intelligence Division, Newton, USA

**Keywords:** In vitro fertilization, Predictive factors, Live birth, Predictive models

## Abstract

**Purpose:**

To dynamically assess the evolution of live birth predictive factors’ impact throughout the in vitro fertilization (IVF) process, for each fresh and subsequent frozen embryo transfers.

**Methods:**

In this multicentric study, data from 13,574 fresh IVF cycles and 6,770 subsequent frozen embryo transfers were retrospectively analyzed. Fifty-seven descriptive parameters were included and split into four categories: (1) demographic (couple’s baseline characteristics), (2) ovarian stimulation, (3) laboratory data, and (4) embryo transfer (fresh and frozen). All these parameters were used to develop four successive predictive models with the outcome being a live birth event.

**Results:**

Eight parameters were predictive of live birth in the first step after the first consultation, 9 in the second step after the stimulation, 11 in the third step with laboratory data, and 13 in the 4th step at the transfer stage. The predictive performance of the models increased at each step. Certain parameters remained predictive in all 4 models while others were predictive only in the first models and no longer in the subsequent ones when including new parameters. Moreover, some parameters were predictive in fresh transfers but not in frozen transfers.

**Conclusion:**

This work evaluates the chances of live birth for each embryo transfer individually and not the cumulative outcome after multiple IVF attempts. The different predictive models allow to determine which parameters should be taken into account or not at each step of an IVF cycle, and especially at the time of each embryo transfer, fresh or frozen.

## Introduction

Numerous parameters are well known to be prognostic factors of IVF success. Over the last two decades, predictive models have been proposed in order to take into account these parameters in their whole. These methodologies have become increasingly more interesting due to (i) the ever-larger amount of data available and (ii) the ever-increasing computational power allowing extraction of valuable information from these large amounts of data. Early works focused on predicting the outcome of IVF treatments in fresh cycles, starting with the direct identification of influencing factors [1] to the simultaneous inclusion of a range of anamnestic couple characteristics [2], every time providing more accurate assessments of the outcomes. Subsequently, predictive models estimated individualized cumulative chances of a first live birth over multiple complete cycles of IVF involving fresh and subsequent embryo transfers [3, 4]. This last model provided live birth predictions after 6 IVF cycles at two time points: the first before undergoing IVF with integration of patient characteristics before treatment; and the second, after completion of the first complete IVF cycle, including fresh and subsequent frozen embryo transfers. More recently, a model was proposed where the probability of live birth after the oocyte retrieval was readjusted with the addition of two parameters, the number of oocytes retrieved and the number of embryos obtained, thereby updating the live birth probability during IVF [5].

The aim of this work is to investigate which and at which stage such potentially predictive factors do in fact help in the estimation of the live birth probability (LBP) at each fresh and frozen cycle transfer. In order to achieve this goal, we created a four-step process to select the statistically significant predictive parameters of the LBP at each step of the IVF procedure.

## Materials and methods

Our approach, although intimately driven by clinical practice, is deeply grounded in data and analytical process via machine learning techniques. Hence, our first step was to gather and curate a database of patients with the entire IVF process of each couple.

### Data

Anonymous data from 5 IVF French centers (3 public and 2 private), including in vitro fertilization (IVF) and intracytoplasmic sperm injection (ICSI) with fresh and subsequent frozen embryo transfers (FET), were retrospectively extracted from January 2014 to December 2017. Cycles with no oocytes retrieved or no embryos obtained were also included. Frozen embryo transfers for which the originated IVF/ICSI cycles were not available were excluded. The rank parameter kept track of the cycle number for each couple, allowing us to properly associate cycles to each returning couple. Donor cycles (oocytes or sperm) and oocytes or embryos cryopreservation cycles were not included.

Data extracted in each participating center are listed in Table 1. An exhaustive phase of data cleansing was performed in order to ensure (i) data uniformity between the 5 centers, (ii) conventions matching, (iii) proper treatment of missing data, and (iv) further manual verifications as necessary. Parameters with more than 10% missing values were removed from the dataset and the “mice” R package was used to impute the remaining missing values, assuming that the missing data were missing at random. Multicollinearity was checked at this step: before running any model, we removed parameters that were strongly correlated (we chose a threshold of 0.8), keeping only one of them. The choice of which variable to keep was driven by the AUC (when there was a measurable impact) and by expertise (when there was not).

**Table 1:**
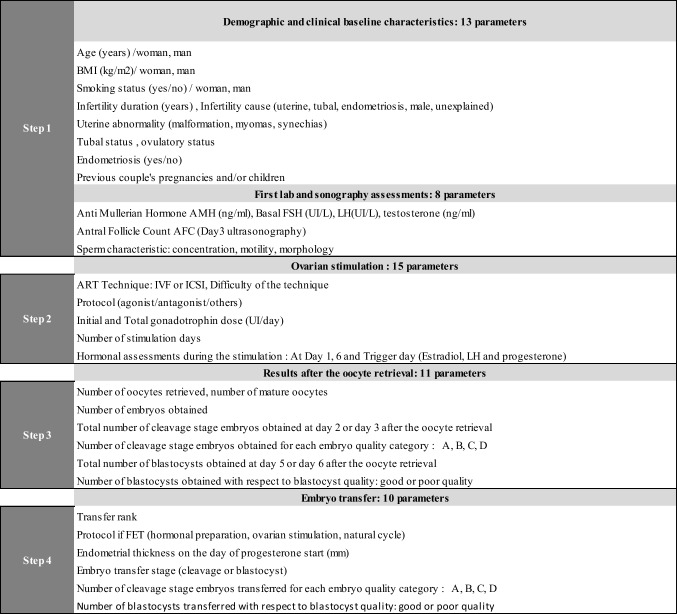
Fifty-seven parameters included in the 4 steps of the IVF process for the development of 4 predictive models

A particular attention was given to homogenize embryo quality grading between participating centers. For simplicity sake, embryos at the cleavage stage were classified into 4 categories. Category A comprised embryos with typical blastomere numbers and less than 10% fragmentation; category B embryos included typical blastomere numbers and less than 30% fragmentation; category C embryos included atypical blastomere numbers and/or between 30 and 50% fragmentation; and category D embryos had more than 50% fragmentation and/or multi-nucleation. For blastocyst grading, the quality assessment was based on Gardner’s classification [6, 7]. Blastocysts were classified into two groups: (1) “good quality” blastocysts (from B3 to B6 with at least one score greater than or equal to “B” for inner cell mass (ICM) and/or trophectoderm) and (2) all other blastocysts (“poor quality” group).

In the dataset, all the numerical parameters could be treated as such, but in some cases, we chose to create categories: age (18–24.9, 25–29.9, 30–34.9, 35–36.9, 37–39.9.9, ≥ 40 years); BMI (< 25, 25–29.9, 30–34.9, ≥ 35 kg/m^2^); antimüllerian hormone, AMH, (< 1, 1–1.5, 1.51–2.5, 2.51–5, ≥ 5.1 ng/ml); antral follicle count, AFC, (≤ 5, 6–10, 11–15, > 15); initial gonadotrophin dose (≤ 150, 151–225, 226–300, > 300 UI per day); and endometrial thickness (< 7, 7–10, > 10 mm). This facilitates the clinical interpretation of the results by aligning them with common practice in the literature.

The end-point chosen to build the predictive models was a live birth event. Data from all 5 centers were subsequently combined to yield a single, anonymized, and reliable database where FET were connected to their corresponding IVF or ICSI.

### Creation of the 4 steps of the IVF process

The database was built to include all the parameters available from the entire IVF process of each patient, from their first infertility consultation to the final embryo transfer (57 parameters in total). This longitudinality of data was divided into four categories, corresponding to the four steps of the IVF process, subsequently corresponding to four iterations of our predictive model (Table 1):

1. Demographic: at the first couple consultation, demographic parameters as well as the first clinical and biological data were collected (1st step) allowing the development of the first predictive model (model 1). Tubal indication in this study included couples for whom a tubal issue was the main problem: uni- or bilateral salpingectomy. Hydrosalpinx were treated surgically prior to IVF process. Cycles with no oocytes or embryos obtained were included at this step.
2. Stimulation: the patients then underwent an ovarian stimulation and corresponding parameters were recorded (2nd step), allowing the development of the second predictive model (model 2). Similarly, cycles with no oocytes or embryos obtained were included.
3. Laboratory: the corresponding parameters were collected after the oocyte retrieval, such as the number of oocytes as well as number and quality of embryos obtained (3rd step), allowing the development of the third predictive model (model 3). Cycles with no oocytes or embryos obtained were excluded at this step.
4. Transfer: finally, for patients achieving an embryo transfer, parameters collected at the embryo transfer step (4th step) allowed the development of the fourth predictive model (model 4). Cycles with no oocytes or embryos obtained were excluded.

The predictive power of the different parameters on live birth probability was calculated at each of these 4 IVF steps. Couples failing to reach a step were no longer included in the subsequent models.

For the subsequent FET, data from steps 1, 2, and 3 were extracted from the originated IVF or ICSI cycles, then the transfer parameters specific to the frozen cycles were added, which allowed the prediction of live birth at each FET (model 4 for FET).

### Multivariate model creation and validation: statistical analysis

The development of the four models followed the recommended TRIPOD checklist for both the methods and the results [8]. LBP was estimated at each IVF step by implementing four generalized logistic regression multivariate models in R (a univariate analysis was also performed for completeness, although the results are not reported here since they are superseded by the multivariate ones). The data were split into training and testing sets over multiple folds. Within each training fold, the models were created using a second fivefold partition and backward selection with cross-validation at each iteration. At the start of the process, all the parameters of the selected category were included, and eventually, only the most predictive ones were selected in the final models. Finally, these models were evaluated over the testing folds, and the performance of the median model is reported here.

The four models resulting from this process were evaluated for their predictive power via the receiver operating characteristic (ROC) curves and associated area under the curves (AUC, or C-statistics). The results presented subsequently include calibration plots, all the adjusted odd ratios of the parameters that survived our selection process (aOR), their 95% confidence intervals (CI), the corresponding ROC curves and associated AUC, and *p*-value for each predictive factor. A *p* < 0.05 was considered to be statistically significant. Note that an aOR equal to one indicates that the corresponding level is the reference in our model.

**Fig. 1:**
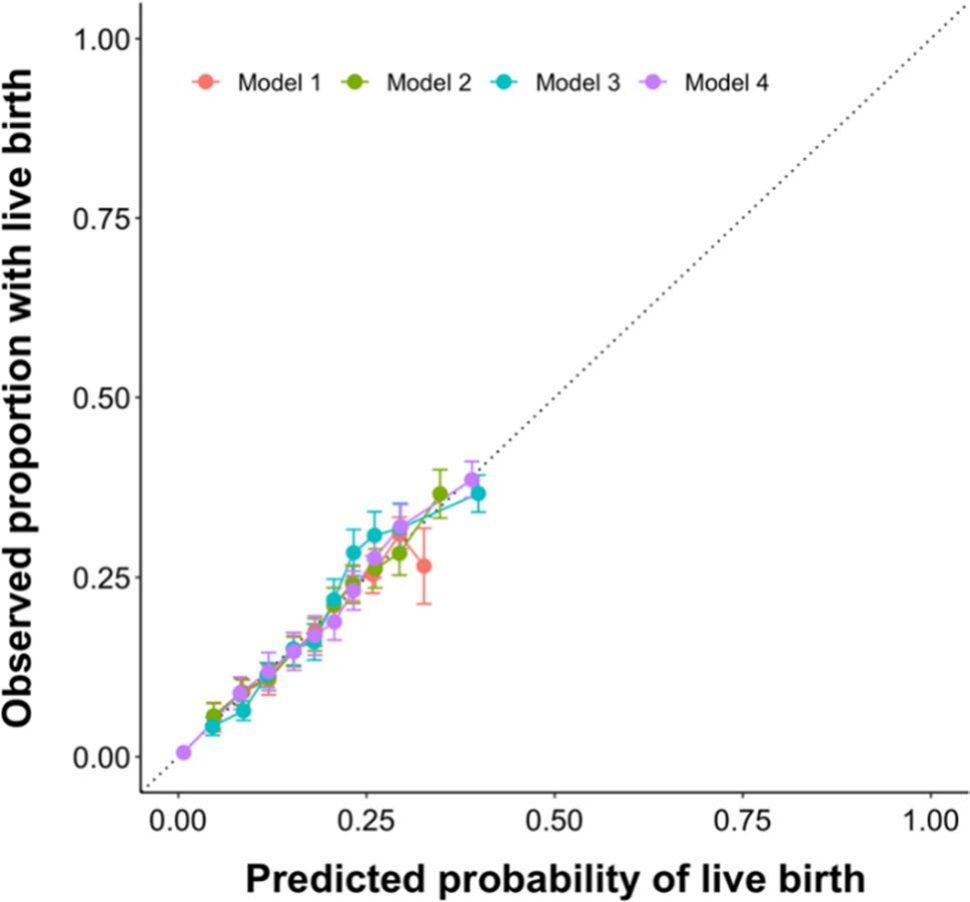
Calibration plots showing the good agreement between the predicted live birth probability and the computed one within each corresponding decile

Calibration plots were created for all four models, summarized in Fig. 1, showing a good agreement between the observed and predicted individual live birth probabilities. The calibration plots were created by splitting the population into deciles and computing the average probability of life birth within each population (Fig. 1).

### Ethical approval

Before undergoing an IVF procedure, patients sign a written consent form allowing anonymous data to be used for retrospective studies. Only patients having given their written consent were included in our study.

The study was approved by our Institutional Review Board (IRB) on the 13th of September 2018.

## Results

A total of 13,574 fresh and 6770 frozen cycles are included in our study, from 8684 couples. The general characteristics of the population provided by the 5 IVF centers are described in Table 2.

**Table 2:**
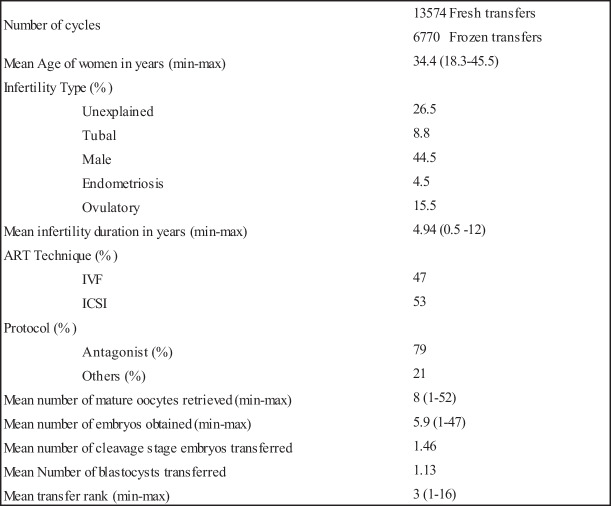
General characteristics and laboratory parameters of the studied population

The global results concerning pregnancy and live birth rates of the studied population are shown in Table 3. In fresh transfers, the overall pregnancy and live birth rates per transfer were 31.7% and 23.2%, respectively. For frozen embryo transfers, pregnancy and live birth rates per transfer were 27.5% and 18.9%, respectively. These results are comparable to those of the French National Registry during the same period [9].

**Table 3:**
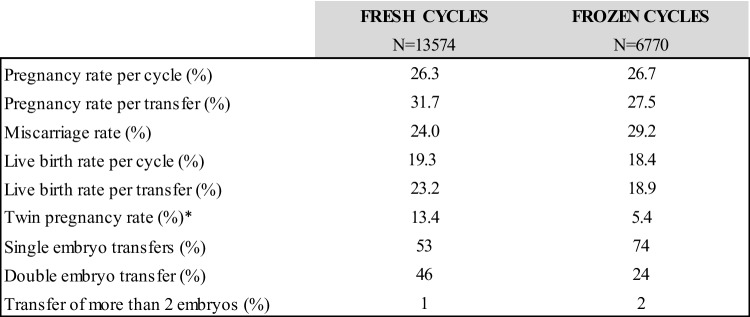
Clinical outcomes in fresh and frozen cycles

^*^Twin pregnancy rate was calculated dividing the number of twin pregnancies by the total number of pregnancies

### Predictive parameters of live birth at each step of the IVF process: fresh transfers

The multivariate odd ratios for all the models and their confidence intervals are presented individually for each model in Tables 4, 5, 6, and 7. Table 8 summarizes the results from all four models and allows the comparison between each model, identifying how the predictive impact of each significant parameter evolves throughout the IVF process.

**Table 4:**
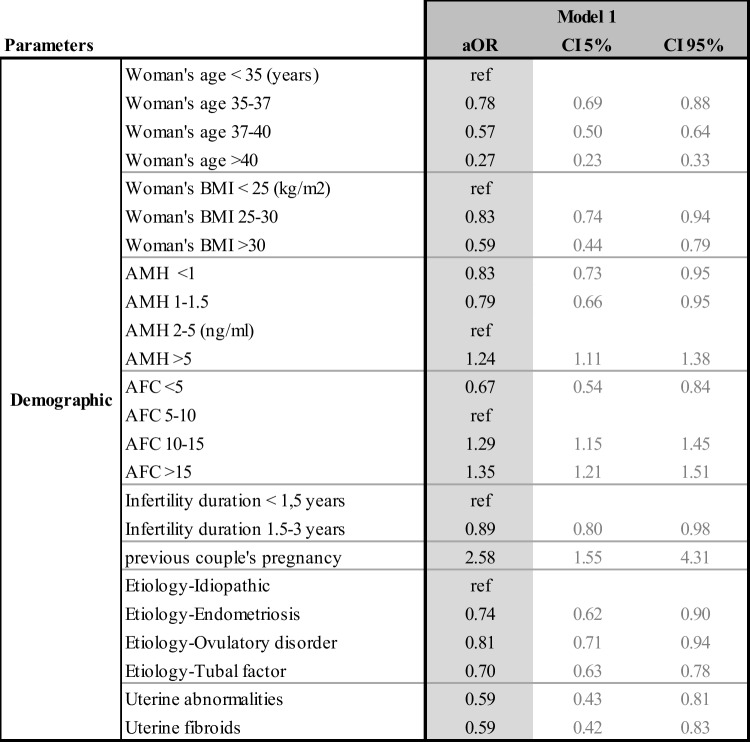
Adjusted odd ratios (aOR) and their confidence intervals (CI) for model 1 statistically significant demographic and initial clinical and biological parameters to predict live birth at step 1

**Table 5:**
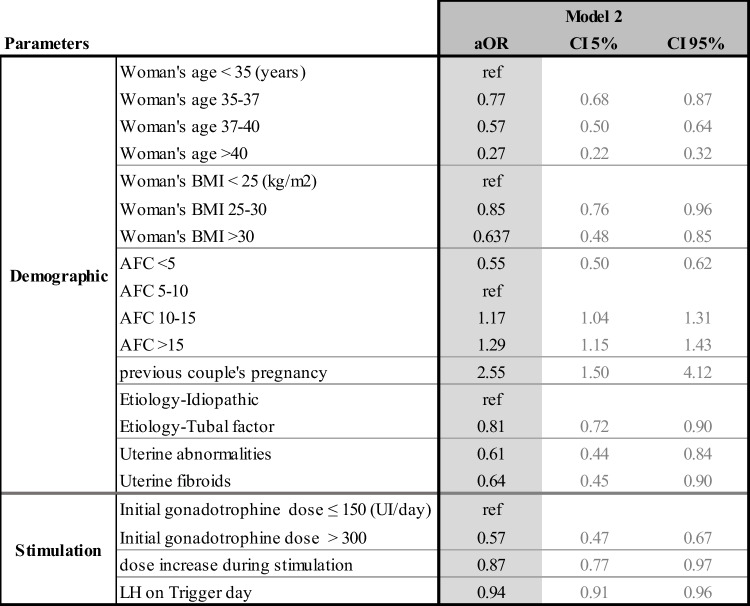
Adjusted odd ratios (aOR) and their confidence intervals (CI) of model 2 statistically significant demographic and stimulation parameters to predict live birth at step 2

**Table 6:**
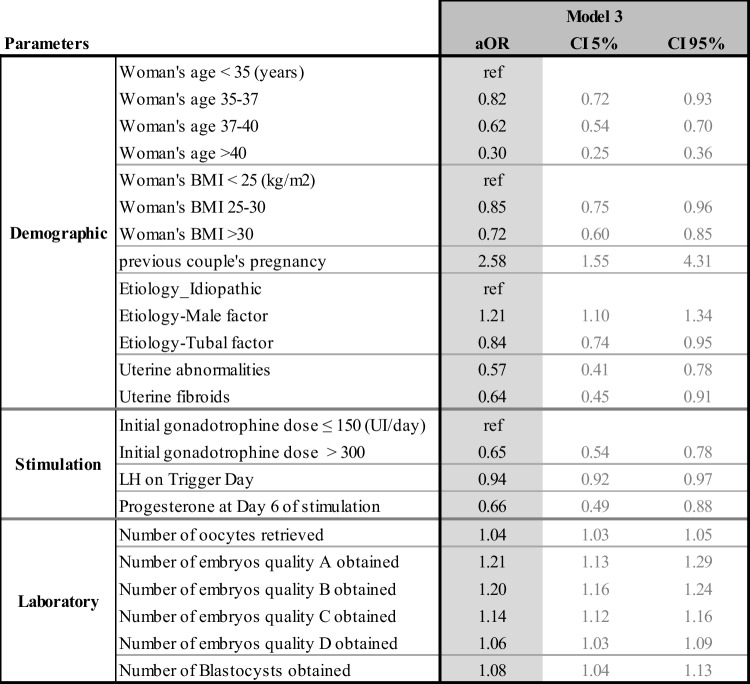
Adjusted odd ratios (aOR) and their confidence intervals (CI) of model 3 statistically significant demographic, stimulation, and laboratory parameters to predict live birth at step 3

**Table 7:**
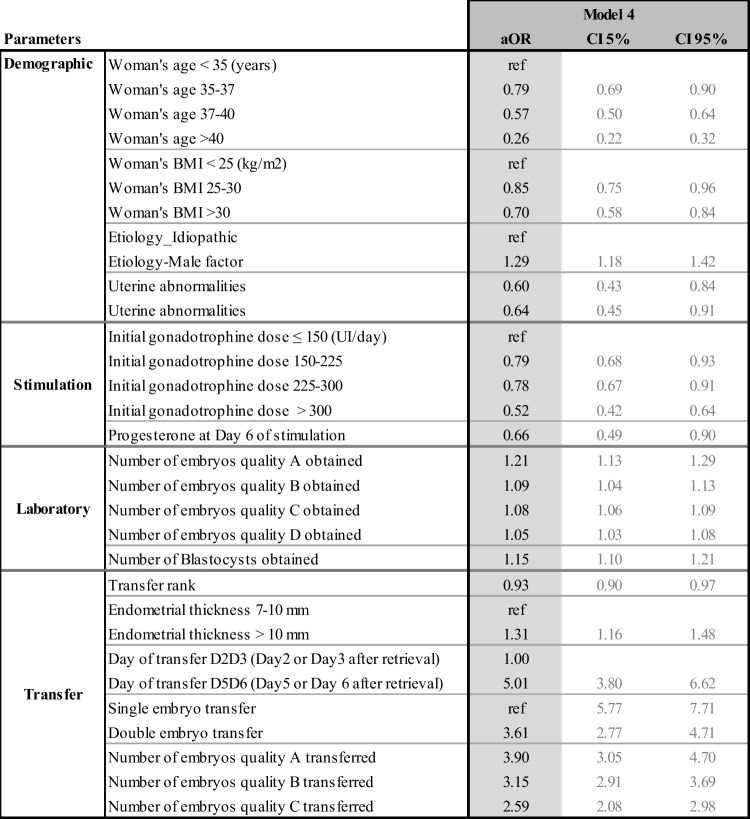
Adjusted odd ratios (aOR) and their confidence intervals (CI) of model 4 statistically significant demographic, stimulation, and laboratory parameters to predict live birth at step 4

**Table 8:**
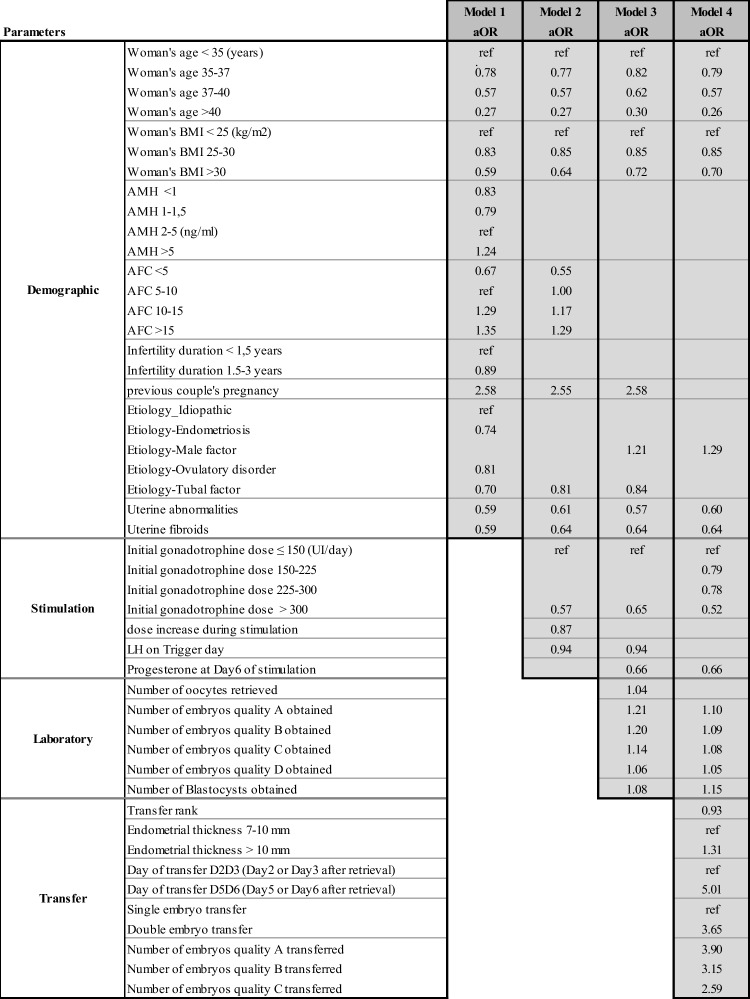
Adjusted odd ratio (aOR) evolution of the selected parameters for all four models (corresponding confidence intervals are provided in Tables 4, 5, 6, and 7)

#### Model 1 (Table 4)

At step 1, after the first consultation, the demographic and the first clinical and biological parameters were used to create model 1. Table 4 shows that 8 parameters were statistically predictive of LB. The odds of live birth decreased with increasing age and woman’s BMI. The ovarian reserve markers predicted lower chances of live birth for women with poor AMH and AFC at this first step. Longer infertility duration also indicated poorer prognosis. Conversely, a history of previous pregnancy in the couple was a good predictor of success (OR = 2.58, 95% CI 1.55–4.31). Aetiology of infertility played a role with lower results for tubal disease (OR 0.70, 95% CI 0.63–0.78), ovulatory disorder (aOR 0.81, 95% CI 0.71–0.94), and endometriosis (OR 0.74, 95% CI 0.62–0.90), as well as the presence of a uterine abnormality (OR 0.59, 95% CI 0.42–0.81).

#### Model 2 (Table 5)

At the second step, ovarian stimulation parameters were added to the demographic ones in order to create model 2. Nine parameters were predictive at this step. Age, BMI, AFC, previous couple’s pregnancy, uterine abnormality, and tubal disease remained predictive. The use of high gonadotrophin doses (> 300 UI/day) and the need to increase the daily dose displayed a negative impact on LBP in fresh transfers. The highest the LH was on the trigger day, the lower was the live birth probability.

#### Model 3 (Table 6)

At the third step, laboratory parameters were added, making a list of 19 possible predictors; 11 remained predictive in this 3rd model, as shown in Table 6. The population with no embryos (1303 cycles) was removed from this model since this information becomes available at this stage. Age, BMI, previous couple’s pregnancy, uterine and tubal abnormality, high gonadotrophin dose, and LH level on triggering day remained continuously predictive for fresh embryo transfers. Progesterone levels on day 6 of the stimulation appeared significant at this step. The number of oocytes, the number and quality of the embryos obtained, and the embryo stage (cleavage or blastocyst) were new predictive parameters of live birth.

#### Model 4 (Table 7)

Finally, at the fourth and last step for fresh embryo transfers, all the parameters available were considered as possible predictors, but only 13 ended up being selected by our process as statistically significant in predicting LBP, yielding model 4. The population with no transferred embryos (1448 cycles) was removed from this model since this information becomes available at this stage. Age, BMI, male factor, uterine abnormality, high gonadotrophin dose, day 6 progesterone, number, quality, and stage of the embryos obtained remained predictive. Most of the transfer parameters also remained predictive: endometrial thickness on the day of progesterone start, quality and stage of the embryo transferred, and transfer rank. The number of embryos transferred appeared significant only for cleavage stage embryos but not for blastocysts. This is explained by the specific clinical practice of all the centers involved in the study, whereby the transfers at the blastocyst stages were almost always single transfers. As a result, the number of blastocysts transferred, almost always 1, ends up having no predictive power and therefore does not appear in our model.

#### Evolutivity of significant parameters (Table 8)

An important implication of our study is that we could go beyond the interpretation of each single model and compare them side by side to see which parameters remain predictive throughout the IVF cycle, to observe the evolution of their impact, or which parameters appear/disappear in the face of other parameters being available. Table 8 is organized for that purpose, allowing the comparison of each model. Hence, Table 8 reveals that woman’s age and BMI show a stable predictive impact on live birth in all four models/steps of the IVF process. Ovarian reserve markers, AMH and AFC, are predictive in the first models, but their predictability disappears as more parameters become known in the successive models. Most causes of infertility are predictive in the early steps of the IVF process but their predictabilities disappear as the IVF process advances. High initial gonadotrophin dose remains negatively correlated with LBP in fresh transfers. Increase in gonadotrophin dose is predictive in model 2 but no longer so in the subsequent models. Lastly, the number of oocytes obtained is a significant predictor in model 3 but not anymore once the number of embryos is known.

**Fig. 2:**
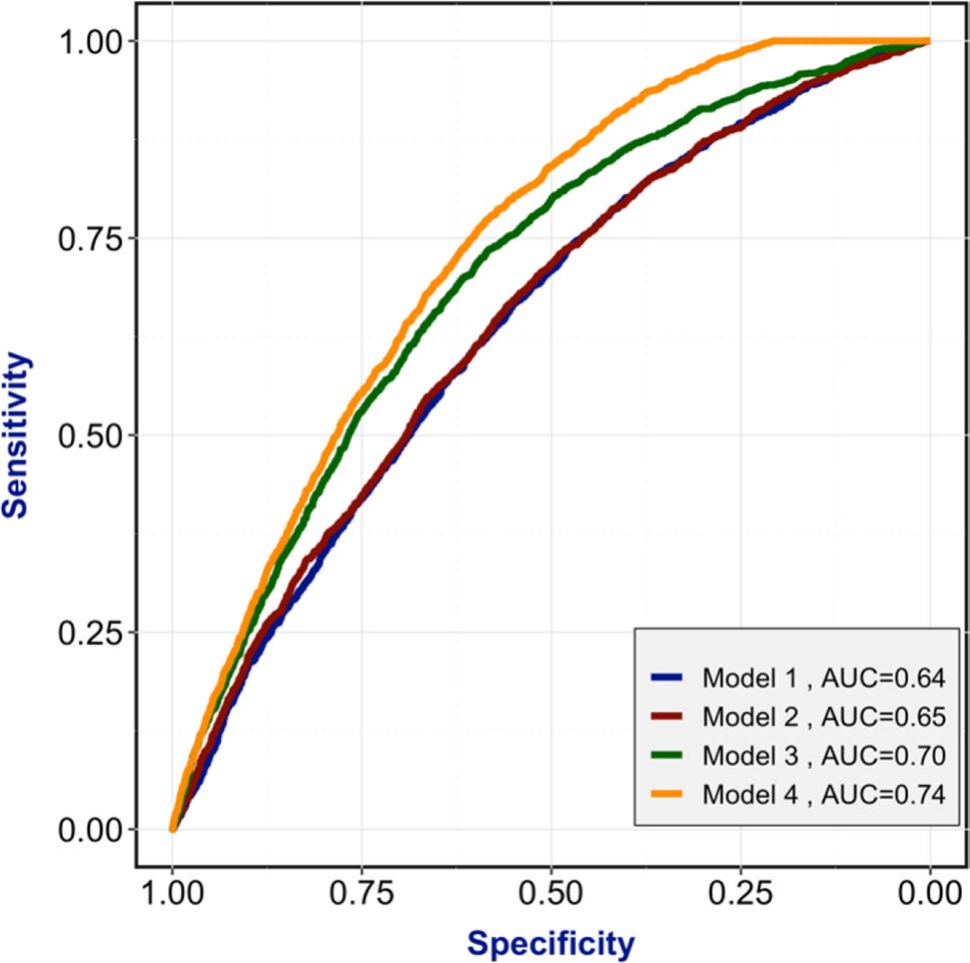
Receiver operating characteristic curve (ROC) for the 4 models for fresh embryo transfers, with their corresponding area under the curve (AUC)

Each of the four models was used to compute a corresponding ROC and AUC, summarized in Fig. 2, allowing a direct comparison of the models’ predictive power. The first curve (AUC = 0.64) corresponds to model 1, using only demographic parameters (step 1). The second (AUC = 0.65), third (AUC = 0.70), and fourth curves (AUC = 0.74) show an increasing predictive power of the models as additional parameters are being added at each step of the IVF process (Fig. 2).

### Predictive parameters of live birth at each step of the IVF process: FET

The same process as presented above was repeated for frozen embryo transfers. Although our population was relatively small, the results obtained were statistically significant. The main difference between fresh and frozen transfers was that the parameters from steps 1, 2, and 3 were already known at the time of the frozen embryo transfer. Consequently, FET models only spanned steps 3 and 4, yielding only two models instead of four. Table 9 summarizes these results and again, should be read both vertically (interpretation of each model) and horizontally (comparing each model).

**Table 9:**
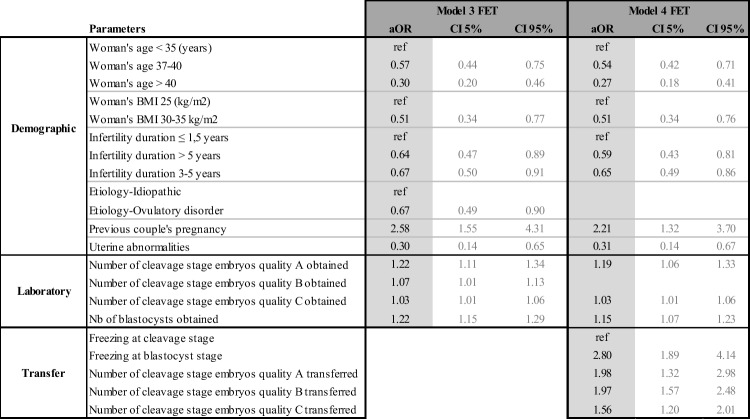
Models for frozen embryo transfers (FET): adjusted odd ratios (aOR) and their confidence intervals (CI) of statistically significant demographic, stimulation, laboratory, and frozen transfer parameters

Table 9 shows that woman’s age at the time of oocyte retrieval, her BMI, infertility duration, uterine abnormality, quality, number, and stage of the embryos obtained remain predictive parameters. Age appears to negatively impact LBP at later years and BMI beyond 30 kg/m^2^. Endometrial thickness, quality, and stage of the embryos transferred are also predictive for FET (Table 9).

**Fig. 3:**
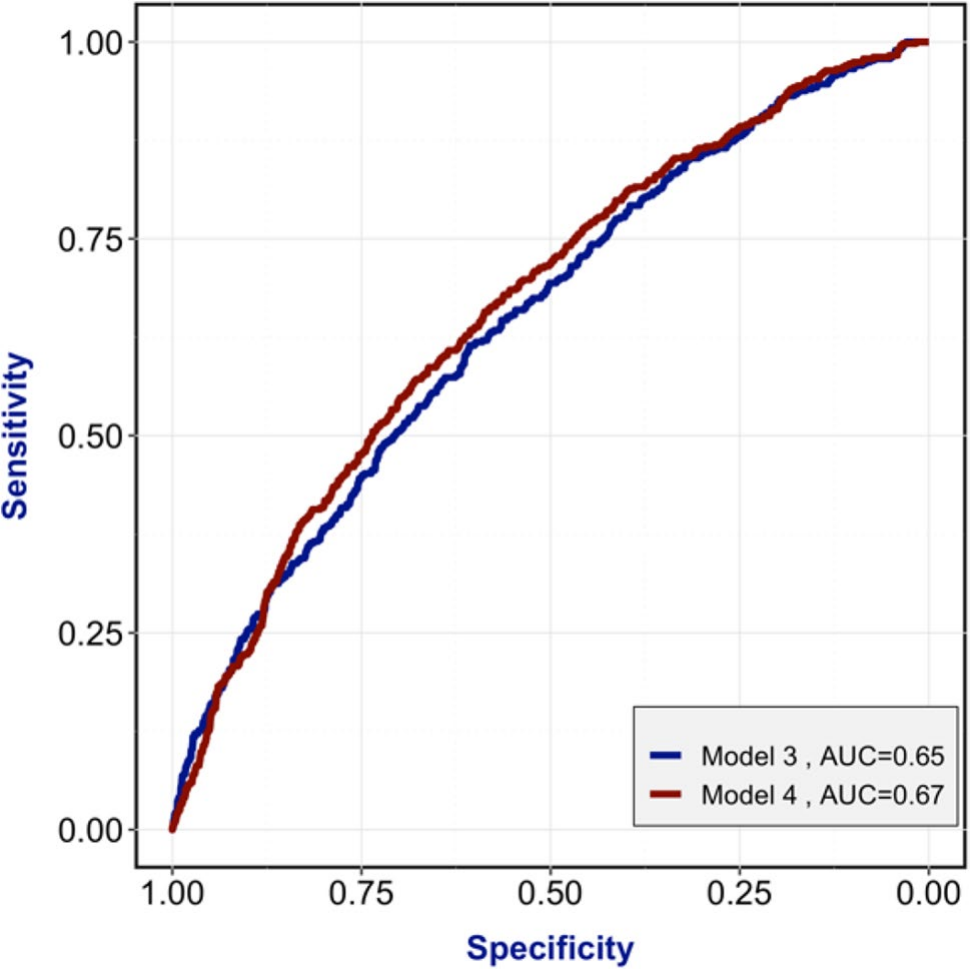
Receiver operating characteristic curve (ROC) for the 2 models for frozen embryo transfers, with their corresponding area under the curve (AUC)

Similar to the fresh transfers, the ROC curves show an improvement in the accuracy of the models to predict live birth after a frozen transfer. The first curve (AUC = 0.65) corresponds to the model including data from the first 3 steps of the IVF cycle (demographic, stimulation, and laboratory data). The second curve (AUC = 0.67) corresponds to the model including FET transfer parameters as well (Fig. 3).

### Examples of how the models can be used to predict live birth probabilities

As an example of the evolutivity of the models, a 40-year-old woman whose baseline characteristics give her 17.2% LBP for her first fresh transfer if her AMH is 2 ng/ml at step 1, has 14.7% recalculated chances if 6 oocytes are retrieved, and 28% if 10 are collected. If that same woman has an AMH of 1.5 ng/ml, her LBP drops to 10% at step 1, but increases to 14.6% and 27% if 6 and 10 oocytes are retrieved, respectively.

**Fig. 4:**
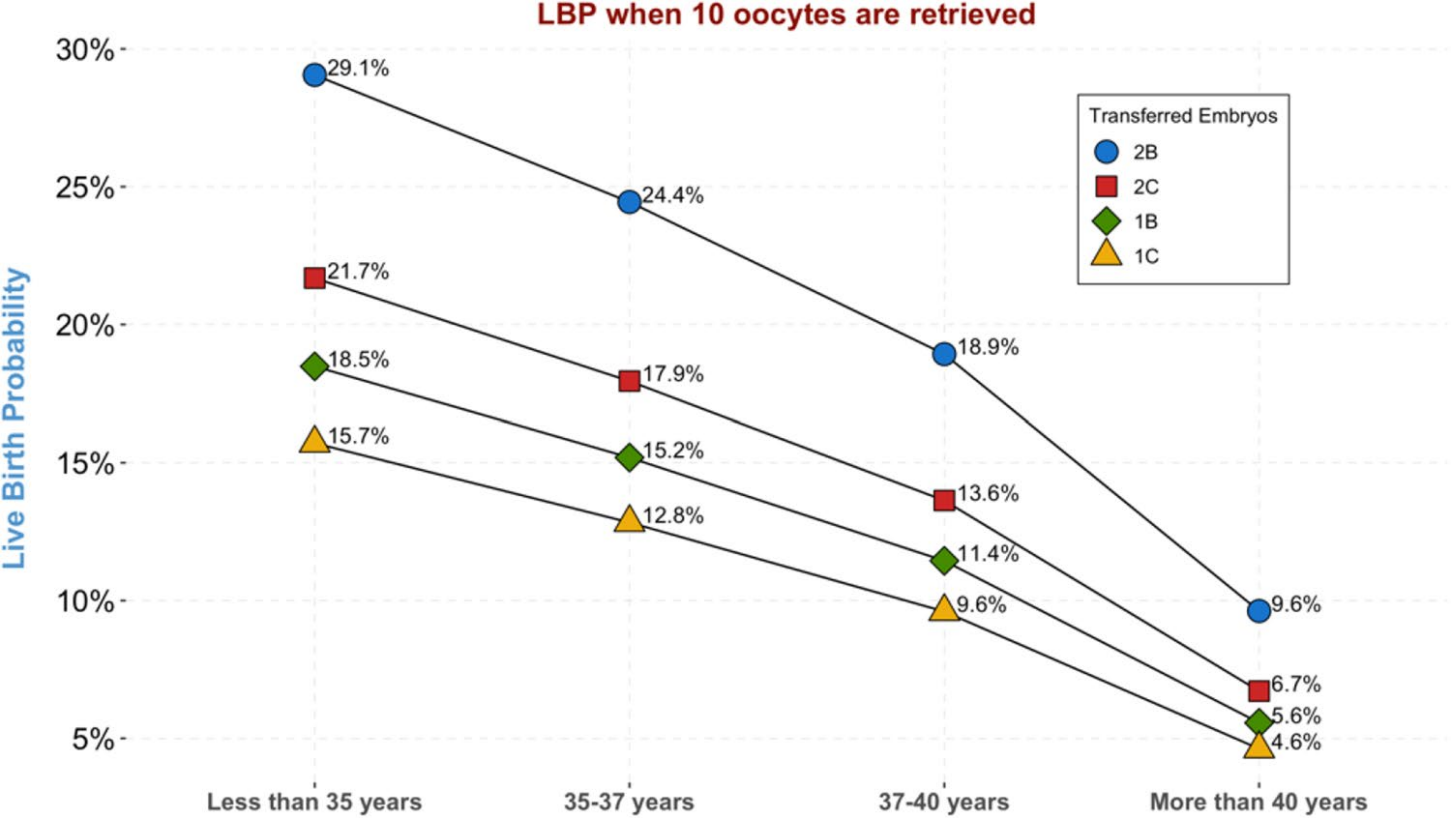
An example of step 4 (transfer step) live birth probability prediction with respect to age and number and quality of cleavage stage embryos to transfer in women having 10 retrieved oocytes. Quality B embryos included typical blastomere numbers and less than 30% fragmentation; C embryos included atypical blastomere numbers and/or between 30 and 50% fragmentation

As another example, Fig. 4 shows how live birth probabilities can be recalculated by the model at the transfer step according to the number and quality of embryos to be transferred in women of different age categories, but with the same steps 1, 2, and 3 characteristics (here, for 10 retrieved oocytes).

## Discussion

Our work determined the predictive factors of live birth that have to be or not taken into account at each step of an IVF cycle. For this purpose, we investigated the largest number of parameters known today, and isolated only the significant ones. Interestingly, we show that the predictive impact of these parameters is not static but evolves throughout the IVF cycle. Some factors that are predictive of live birth at the first step of the IVF attempt (at the time of the first infertility evaluation) are no longer predictive at later steps due to the integration of other factors. Moreover, some couples may fail at any step of the treatment, so the study population evolves for each model. Additionally, the live birth probabilities were determined for each embryo transfer, fresh or frozen, while most of the published predictive models assessed live birth chances after the first fresh transfer, or provide cumulated probabilities at the end of a complete IVF journey [1, 2, 10–14].

The integration of all couples’ available parameters into each model reveals those predictive at each step, and how their predictive impact evolves.

As expected, we observe women’s age as being the most determining factor at each step of the IVF process for fresh transfers [15]. Interestingly, however, for FET, the predictive impact of women’s age at the time of embryo freezing appears at a more advanced age than for fresh transfers. Receiving at least one FET is conditional upon having at least two good quality embryos (except for the few cases of all freeze processes of only one embryo) and selecting better prognosis patients, possibly explaining the delayed impact of age on live birth in FET.

The results also show a stable negative impact of female obesity on the LBP for fresh transfers. This effect on IVF results is well documented, probably due to an impaired endometrial receptivity [16–19]. However, for frozen embryo transfers, only BMIs beyond 30 kg/m^2^ impact chances of live birth in our study. The literature regarding this subject is still conflicting: some authors published lower results in FET in a freeze all policy in overweight women whereas a recent study found similar live birth rates after frozen blastocyst transfers for obese patients as compared to normal weight patients [20, 21].

In FET, we observe that female age and BMI have the strongest impact when pushed to their higher ranges, older or obese women. We hypothesize that having cryopreserved embryos translates into better oocyte or embryo quality, pushing back the limit of age and BMI where these parameters impact live birth. However, future properly designed studies are needed to confirm this hypothesis. In this study, male’s age and BMI did not appear as significant predictive factors.

Our results also show that ovarian reserve markers, AMH and AFC, are predictive of live birth before the number of oocytes is known, but no longer after the oocyte retrieval. AMH is well-known to be strongly associated with ovarian response and oocyte yield [22], but its ability to predict live birth remains conflicting. Indeed, the studies reporting AMH as an independent predictive factor of live birth in fresh and frozen embryo transfers have only found a poor predictive accuracy [23–25], not yielding any additional value on top of age [26]. Our results are in agreement with a recent publication concluding that AMH is a good biomarker for oocyte quantity but not for oocyte quality [27]. Additionally, we show that when the number and quality of the embryos obtained becomes known, the number of oocytes retrieved is no longer predictive of live birth. This confirms the importance of the embryo cohort on chances of live birth for each transfer.

Our results also show a strong impact of the daily and total gonadotrophin doses used for the ovarian stimulation after fresh embryo transfers. Model 3 shows an OR of 0.65 (95% CI 0.54–0.78) for women receiving more than 300 UI/day as compared to those receiving ≤ 150 UI/day. This negative impact has also been demonstrated in more than 650 000 ART cycles with a decreased live birth rate in fresh transfers with increasing total FSH dose [28, 29]. Notably, however, this negative impact was not found in the model for the frozen embryo transfers. These results support the hypothesis of a deleterious effect of high gonadotrophin doses on endometrial receptivity [30]. Increasing FSH doses appears also deleterious while duration of ovarian stimulation did not appear as a significant factor.

Concerning progesterone, a negative effect of high progesterone level on stimulation day 6 in fresh transfers was observed in our models, suggesting a known deleterious effect of premature progesterone elevation on endometrial receptivity [31]. The deleterious effect of high progesterone level on the trigger day has been well documented [32, 33]. We did not observe this negative impact on trigger day because all the patients with a progesterone ≥ 1.5 ng/ml on the hCG day benefit from a freeze all approach in the participating centers.

Recent reviews show that numerous predictive models, most concerning fresh cycles, exist today in reproductive medicine as a counselling tool to inform patients on their chances of live birth [34–36]. However, such adaptive models like ours might also have beneficial purposes for couples. For instance, illustrating the modification in live birth probability associated with changes in lifestyle might help couples initiate weight loss strategies or refrain from smoking. This could be explored in further prospective studies.

Contrary to the first predictive models, the latest tends toward readjusting live birth probabilities at different time points of the IVF process. The last published model aimed to predict cumulative live birth after three complete IVF cycles at two time points: before starting the first IVF cycle and after the first complete IVF cycle, including FET, just before starting the second cycle; the aim of these models is to allow couples to make the most informed decision possible [3–5, 13, 37]. Our work evaluates live birth chances individually for each embryo transfer and not after multiple IVF attempts. The objective is to determine the most predictive parameters that have to be taken into account at each step of the IVF process and for each transfer.

The accuracy of LBP increases with the addition and reintegration of all the numerous available couples’ parameters at each step of the IVF process. Additionally, this very highly curated database ensures that all the information used is complete. The main limitation of our work remains the limited number of participating centers and therefore the limited number of cycles included. Our model was developed using IVF data from French centers exclusively. Since the infertile population in France might be slightly different than in other countries (i.e., BMI, ethnicity, smoking status…), our models would benefit from being confirmed by external international validation, which is planned in a future work [38].

The objective of our models is to determine in daily practice the important influencing parameters at different time points, and especially at the time of the embryo transfers.

To conclude, these evolutive models allow the identification of parameters that are predictive or not of live birth and that clinicians could take into account at the different steps of IVF, and for each embryo transfer, fresh or frozen.
