# Correction to: Adaptive data-driven models to best predict the likelihood of live birth as the IVF cycle moves on and for each embryo transfer

**DOI:** 10.1007/s10815-023-02717-y

**Published:** 2023-01-18

**Authors:** Véronika Grzegorczyk-Martin, Julie Roset, Pierre Di Pizio, Thomas Fréour, Paul Barrière, Jean Luc Pouly, Michael Grynberg, Isabelle Parneix, Catherine Avril, Joe Pacheco, Tomasz M. Grzegorczyk

**Affiliations:** 1 Department of Assisted Reproductive Technology and Fertility Preservation, Clinique Mathilde, Service Assistance Médicale À La Procréation, 76100 Rouen, France; 2 Nantes Université, Inserm, Centre de Recherche en Transplantation Et Immunologie, UMR 1064, ITUN, 44000 Nantes, France; 3 Service de Médecine Et Biologie du Développement Et de La Reproduction, Nantes, France; 4 Centre de Procréation Médicalement, Assistée Hôpital Estaing-CHU de Clermont Ferrand, 63003 Clermont Ferrand, France; 5 Department of Reproductive Medicine and Fertility Preservation, Hôpital Antoine Béclère, Hôpitaux Universitaires Paris Sud, Assistance Publique-Hôpitaux de Paris, 92140 Clamart, France; 6 Department of Reproductive Medicine & Fertility Preservation, Hôpital Jean Verdier, 93140 Bondy, France; 7 Université Paris-Sud, Université Paris Saclay, Le Kremlin Bicêtre, 93140 France; 8 Department of Cytogenetic and Reproductive Biology, Hôpital Jean Verdier, 93140 Bondy, France; 9 Unité Inserm U1133, Université Paris-Diderot, 75013 Paris, France; 10 Centre AMP Eurofins Fertilité Jean Villar, Bordeaux, France; 11 Teranalytics, Artifcial Intelligence Division, Newton, USA

**Correction to: Journal of Assisted Reproduction and Genetics**

**https://doi.org/10.1007/s10815-022-02547-4**

The “Funding” information section was missing from this article and should have read “This work was supported by the 2018 FORWARD grant (FORWARD2018_3) from GEDEON RICHTER.”

The original article has been corrected.

